# Role of Different Material Amendments in Shaping the Content of Heavy Metals in Maize (*Zea mays* L.) on Soil Polluted with Petrol

**DOI:** 10.3390/ma15072623

**Published:** 2022-04-02

**Authors:** Mirosław Wyszkowski, Natalia Kordala

**Affiliations:** Department of Agricultural and Environmental Chemistry, University of Warmia and Mazury in Olsztyn, Łódzki 4 Sq., 10-727 Olsztyn, Poland; natalia.kordala@uwm.edu.pl

**Keywords:** petrol contamination, material amendments, heavy metals, *Zea mays* L.

## Abstract

Petroleum substances are among the xenobiotics that most often contaminate the natural environment. They have a strong effect on soil, water, and other components of the environment. The aim of this pot experiment has been to determine the effect of different soil material amendments (compost, 3%; bentonite, 2% relative to the soil mass or calcium oxide, in amounts corresponding to one full hydrolytic acidity) on the content of heavy metals in aerial parts of maize (*Zea mays* L.) grown on soil polluted with petrol (0, 2.5, 5, and 10 cm^3^ kg^−1^ of soil). The content of all heavy metals, except copper, in the aerial biomass of maize was positively correlated, but biomass yield negatively correlated, with the increasing doses of petrol. The highest increase in the content of heavy metals was noted for chromium and manganese. Materials used for phytostabilisation (compost, bentonite, and calcium oxide) had a significant effect on the content of heavy metals and biomass yield of maize. They contributed to the modified accumulation of elements, especially chromium, copper, and cobalt in the aerial biomass of maize. In comparison with the control series (without material amendments), the application of calcium oxide proved to be most effective. It had the most evident influence on the chemical composition of maize, limiting the accumulation of lead, zinc, manganese, and iron and increasing biomass yield.

## 1. Introduction

For centuries, human activity has been causing changes in the environment, modifying its natural characteristics and polluting it with hazardous and toxic substances [1,2]. Petroleum compounds are among the xenobiotics that most often contaminate the natural environment [3]. This type of a contaminant reduces or completely destroys the fertility of soil [4], changes the elemental composition of soil and its enzymatic properties [5,6], and reduces yields of agricultural crops [7,8,9]. In addition, it leads to the loss of aesthetic values of ecosystems, their desertification, and to the secondary pollution of groundwater and air [10,11]. It also inhibits or totally prohibits the development of soil microorganisms [12,13,14].

Petroleum hydrocarbons pose a threat to the functioning of ecosystems in polluted areas and to the life of animate organisms due to their mutagenic, carcinogenic, and embryotoxic properties [15]. Due to their hydrophobic character and resistance to degradation, they can accumulate in soil and in subsequent links of trophic chains [16].

Soil is the most important natural resource, which plays a significant role in the flow of matter and energy. It also conditions the growth of plants, thereby affecting the production of food [17]. Soil pollution is closely linked to the developing industries, progressing urbanisation, and to agriculture and mining. Petroleum substances are the most common soil pollutants [18] due to the widespread use of petroleum products. Crude oil and its derivatives permeate into the natural environment mostly during their storage and transport [19,20]. Severe soil contamination with petroleum products causes a decrease in biodiversity, inhibition or arrest of plants’ growth and development, and a high risk of migration of pollutants, mainly due to wind or water erosion. The spread of these pollutants can disturb biogeochemical cycles and intensify the risk of groundwater pollution [21].

However, petroleum-derived substances can support the growth of plants when they act as a source of nutrients absorbed by plants for their development (e.g., carbon, hydrogen, oxygen, and nitrogen), but this can happen as long as their content does not exceed 1 mg kg^−1^ of soil [18]. When present in higher concentrations, they become toxic to plants. Their adverse influence is multi-directional. Once they are absorbed by plants, they can modify the permeability of cellular membranes [22], inhibit the mitotic activity of the root’s meristem [23] or induce stress by changing the water and oxygen relations [24]. Moreover, the soil contamination with petroleum derivatives decreases the soil content of organic matter and mineral elements and compounds available to plants, such as phosphorus, potassium, sulphates, and nitrates [25,26].

Soil contamination with petrol causes depressed yields of aerial parts of plants [27], alters the chemical composition of plants [28,29], and has a negative impact on the nitrification process in soil. As demonstrated by Kucharski et al. [30], the oxidation of ammonium cations decreases by 88% in the presence of petrol, and fertilizer nitrogen is subject to strong immobilisation.

Effective ways in the reclamation of contaminated soil are sought and developed in order to protect the natural environment. There are several remediation technologies applied to such soils whose aim is to contain, immobilise, and remove pollutants using physical, chemical, and biological processes [31]. One of the solutions consists of the application of different mineral and organic materials in the phytostabilisation process, which have a positive effect on the properties of soil and growth of plants [32,33]. Numerous experiments have demonstrated the beneficial influence of charcoal on the growth and development of oats [34], calcium oxide and compost on the yield of maize [35], or calcium oxide on the yield of straw of wheat [36].

The introduction of bentonite, calcium oxide, and compost to soil enables the effective immobilisation of pollutants and helps to restore the biological balance of soil contaminated with petroleum products [37]. Bentonites are clay rocks composed mainly of the smectite group minerals (containing no less than 75% of montmorillonite) [38]. When added to soil, they increase the soil’s sorption capacity and its reaction, thereby decreasing amounts of phytoavailable forms of heavy metals in soil [39,40]. Compost raises the pH and the organic matter content of soil, resulting in the immobilisation of heavy metals as they are bound in insoluble complex compounds [41,42]. In turn, lime is the material most often used in agriculture to neutralise the soil reaction, improve conditions for the growth of plants, and to reduce the uptake of heavy metals by plants [43]. Its application reduces the pool of the mobile fractions of heavy metals by their absorbing, complexing, or precipitating the insoluble phases after the soil’s chemical properties have been changed, including its higher pH [44,45].

In the current study, we hypothesized that the application of material amendments (compost, bentonite, and calcium oxide) to the soil would limit the negative effect of soil pollution with petrol on biomass and the content of heavy metals in maize (*Zea mays* L.). This led to detailed predictions that: (1) petrol effect on the aerial mass of maize would be negative, with increased content of heavy metals in the biomass of maize; (2) the application of material amendments to the soil would limit the effect of petrol contamination on plants; and (3) mineral amendments would have a greater effect than compost on the content of heavy metals and biomass of maize.

## 2. Materials and Methods

### 2.1. The Methodological Assumptions of the Research

The research was based on a pot experiment carried out in a greenhouse at the University of Warmia and Mazury in Olsztyn (north-eastern Poland). The soil to fill in the pots originated from the humus horizon of Eutric Cambisol formed from sandy loam [46]. It had the following properties: pH_KCl_—5.54; hydrolytic acidity (HAC)—23.2 mmol (+) kg^−1^; total exchangeable bases (TEB)—107.0 mM (+) kg^−1^; cation exchange capacity (CEC)—130.2 mM (+) kg^−^^1^; base saturation (BS)—82.2%; content of C_org_—6.34 g kg^−1^, content of available phosphorus—29.32 mg kg^−1^, potassium—51.78 mg kg^−1^, and magnesium—62.48 mg kg^−1^. The content of heavy metals in the soil is presented in Table 1. This was a two-factorial experiment, where the first order factor consisted of increasing doses of unleaded petroleum 95: 0, 2.5, 5 and 10 cm^3^ kg^−1^ of soil. The second order factor was the application of compost (3%), bentonite (2% relative to the soil mass), and calcium oxide (50% CaO) to soil in amounts corresponding to one full hydrolytic acidity (1.08 g kg^−1^ of soil). These materials were added to soil in order to constrain potentially negative effects of petrol on plants. Compost was composed of waste from a farm, such as deciduous tree leaves (maple, apple, cherry, and plum trees), cattle manure, and garden peat. Compost was composted for 6 months. Calcium oxide was characterized by the largest content of manganese, cadmium, chromium, nickel and cobalt, bentonite—lead and iron, in turn, compost—zinc, and copper. In addition, compost contained the least cadmium, lead, chromium, nickel, manganese, iron and cobalt, and calcium oxide—zinc and copper (Table 1). The amount of different material amendments and petrol doses were determined based on previously made research. Soil was also enriched with macro- and micronutrients in the same doses throughout the experiment (in mg kg^−1^ of soil): N—25 CO(NH_2_)_2,_ P—30 (KH_2_PO_4_); K—70 (KH_2_PO_4_ + KCl); Mg—50 (MgSO_4_·7H_2_O); Mn—5 (MnCl_2_·4H_2_O); Mo—5 [(NH_4_)_6_Mo_7_O_24_·4H_2_O]; B—0.33 (H_3_BO_3_). While setting up the experiment, petrol, compost, bentonite, and calcium oxide, as well as the macro- and micronutrients were mixed manually and very exactly with a 9 kg batch of soil, and then transferred to polyethylene pots. Maize (*Zea mays* L.) of the cultivar Scandia was the tested crop. Maize is one of the most widely cultivated plant species in the world and in our country. Constant soil moisture was maintained throughout the entire experiment (60% of water holding capacity) The experiment was conducted with four replications. Maize was harvested at the tasselling stage, which is when aerial parts (leaf and stems) plant samples for laboratory analyses were taken.

### 2.2. Methodology of the Laboratory and Statistical Analyses

Samples of maize aerial parts were dried at a temperature of 60 °C and ground. The plant samples were wet mineralized in 65% concentrated nitric acid (HNO_3_ p.a. of the density 1.40 g cm^−3^) in Teflon^®^ Xpress vessels, in a microwave digestion system MARS 6-CEM Corporation (Matthews, NC, USA) according to the methodology US-EPA3051 [47]. The content of heavy metals (cadmium, lead, chromium, nickel, zinc, copper, manganese, iron, and cobalt) was determined on a SpectrAA 240FS spectrophotometer (Varian Inc., Mulgrave, Australia) with atomic absorption spectrophotometry [48].

Prior to the experiment, soil samples (1 kg) were taken from the full depth of the humus horizon. Soil samples were air dried and sieved. An analysis of the basic soil properties was made, where the following were determined: soil reaction (pH) with the potentiometric method in an aqueous solution of KCl in the concentration of 1 M dm^−3^ [49]; hydrolytic acidity (HAC) and total exchangeable bases (TEB)—with the Kappen method [50]; and the contents of available phosphorus and potassium with the Egner–Riehm method [51], and of available magnesium with the Schachtschabel method [52]. Based on the hydrolytic acidity (HAC) and total exchangeable bases (TEB), the total cation exchange capacity (CEC) and base saturation (BS) were calculated from the formulas: CEC = TEB + HAC; BS = TEB·CEC^−1·^100 [48]. The digested soil samples were analysed to determine the total content of heavy metals using flame atomic absorption spectrometry (FAAS) with an air–acetylene flame after wet-digestion in a mixture of concentrated hydrochloric acid and nitric acid in a MARS 6 microwave digestion system (CEM Corporation, Matthews, NC, USA), according to the method US-EPA3051 [48].

The quality of heavy metals analyses was monitored using the certified material from the Chinese National Analysis Centre for Iron and Steel, Beijing, China (NCS ZC 73030) for plants and CRM Soil S-1 from the AGH University of Science and Technology in Kraków, Poland, for soil and the standard solutions by Fluka (Cd 51994, Pb 16595, Cr 02733, Ni 42242, Zn 188227, Cu 38996, Mn 63534, Fe 16596, Co 119785.0100).

The data distribution normality of the data was verified with the Kruskal–Wallis and Shapiro–Wilk tests. The research results underwent statistical analysis in Statistica [53] using a two-factorial analysis of variance ANOVA and Honestly Significant Difference (HSD) Tukey test. Homogeneous groups were calculated at a level of significance of *p* ≤ 0.01, *n* = 48, principal component analysis (PCA) and percentage of observed variation using the η2 coefficient in the ANOVA approach.

## 3. Results

The influence of soil contamination with petrol and of the application of compost, bentonite and calcium oxide on the content of heavy metals in the aerial biomass of maize and biomass yield were varied (Table 2, Table 3 and Table 4).

### 3.1. The Impact of Petrol Contamination on Plants

In the series without the addition of neutralising materials, the increasing doses of petrol caused a significant increase in the content of chromium and manganese in the aerial parts and decrease biomass yield of maize (Table 2, Table 3 and Table 4).

Compared to the control (not contaminated with petrol), the content of these elements in maize at the highest soil pollution level (10 cm^3^ kg^−1^) decreased almost two-fold in biomass yield and increased more than three-fold and three-fold, respectively, in chromium and manganese. High doses of petrol also increased the content of lead by 38%, nickel by 62%, iron by 41%, and cobalt by 43% in the aerial organs of maize relative to the control. In the same series, the soil contamination with petrol contributed to the reduction of the copper content in maize by 33% relative to the control. No significant effect of this factor on the content of cadmium or zinc was observed.

### 3.2. The Impact of Materials Application on Plants on Soil Contaminated with Petrol

Regardless of the degree of soil pollution, all the materials (compost, bentonite, and calcium oxide) added to soil in order to alleviate possible negative effects of the petrol contamination significantly modified the average content of heavy metals in the aerial parts and biomass yield of maize (Table 2, Table 3 and Table 4). The neutralising effect depended on the dose of petrol. The application of compost, bentonite, and calcium oxide contributed to an increase in the average content of cadmium, chromium, copper, and cobalt in the aerial organs and biomass yield of maize, while significantly limiting the accumulation of manganese. The application of calcium oxide proved to be most effective. In the series where soil was polluted with the highest dose of petroleum (10 cm^3^ kg^−1^), calcium oxide led to a three-fold reduction in the content of manganese as well as reducing the content of lead (by 19%), zinc (by 23%), and iron (by 24%) in maize in comparison with the control variant. The correlation was reverse for cadmium, chromium, copper, cobalt, and biomass yield. The application of CaO to soil in the same objects was conducive to the accumulation of these four elements. The biggest changes were noted in the case of copper and chromium, the concentrations of which in maize aerial parts were twenty-three-fold and over two-fold higher, respectively, than in the control treatment.

Soil remediation with bentonite led to an elevated accumulation of all heavy metals in the maize plants, except lead, manganese, and iron (Pb, Mn, and Fe) in the objects polluted with the highest petrol amount (Table 2, Table 3 and Table 4). The results obtained after the application of compost were less unequivocal and consistent. When compost was added to soil polluted with a dose of petrol equal 10 cm^3^ kg^−1^, the maize harvested from this soil was found to contain more chromium, copper, and cobalt in its aerial parts, while having less manganese (by 51%) and iron (by 21%). Similar relationships were determined in the series not contaminated with petrol. Compost had no statistically significant effect on the content of zinc in the aerial biomass of maize.

**Figure 1:**
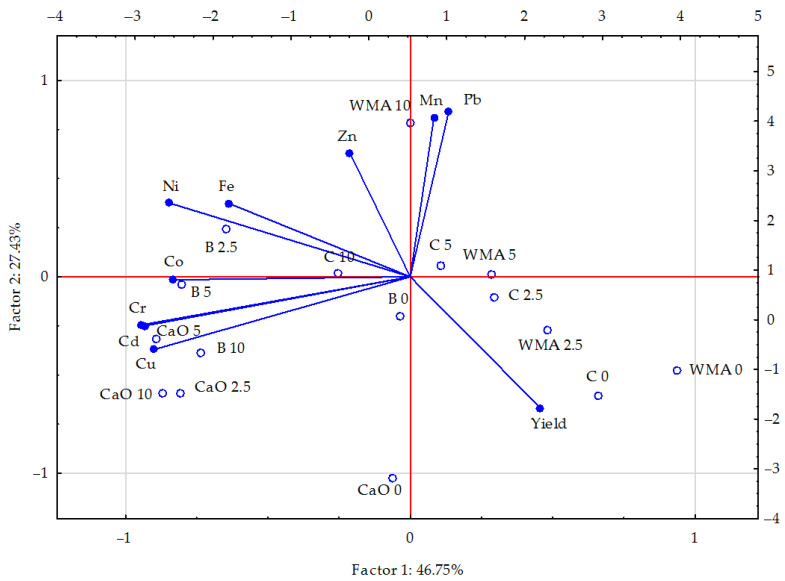
Content of heavy metals in the aerial parts of maize (*Zea mays* L.) illustrated with the PCA method. Key: vectors represent analysed variable (content of Cd, Pb, Cr, Ni, Zn, Cu, Mn, Fe, and Co; left, Y, and bottom, X axis), points show the samples with elements (WMA—without material amendments, C—compost, B—bentonite, and CaO—calcium oxide; 0—0 cm^3^ (control), 2.5—2.5 cm^3^, 5—5 cm^3^, and 10—10 cm^3^ petrol per kg of soil; right, Y, and top, X axis).

Based on the vector variables (Figure 1), the cumulative influence of soil pollution with petrol and the phytostabilisation process on the chemical composition of maize was revealed. The strongest positive correlation was noted for copper versus cadmium and chromium, and for manganese versus lead. The strongest negative correlation appeared between copper versus manganese, and lead, nickel, and cobalt versus yield, while a weaker one was found between lead versus cadmium and chromium. The PCA showed that the correlation of the set of data for the first group of heavy metals (cadmium, chromium, nickel, copper, iron, and cobalt) was 46.75%, while for the other group (lead, zinc, manganese, and maize biomass yield) it equalled 27.43% of a variance. When lengths of the vectors of the analysed elements were compared, it turned out that the vectors of copper, cadmium, nickel, chromium, and biomass yield were longer than the vectors of the other elements, which implicates their greater contribution to the correlation of the data set.

The scattering of points in Figure 1 suggests that the application of the soil reclamation materials (especially calcium oxide) tended to have a positive influence, reducing the content of the analysed heavy metals in the aerial biomass of maize. Calcium oxide and, to a lesser extent, bentonite (in the case of the highest contamination with petrol) had the greatest effect on the content of copper, cadmium, and chromium (positive correlation) as well as manganese and lead (negative correlation) in maize.

**Figure 2:**
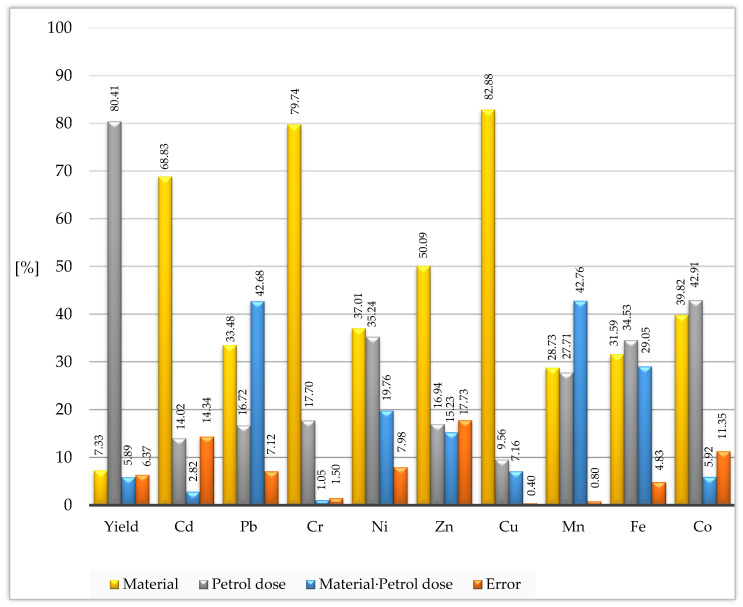
Percent contribution of variable factors according to the yield and content of heavy metals in aerial parts of maize (*Zea mays* L.).

Determination of the percentage of observed variation with the help of the η2 coefficient and using the ANOVA method demonstrated that the content of heavy metals in maize more depended on the type of a neutralizing material added to soil than petrol dose. This effect constituted 37.01%, 50.09%, 68.83%, 79.74%, and 82.88% of the share of a given variable for nickel, zinc, cadmium, chromium, and copper, respectively (Figure 2). Much lower values were determined for the remaining elements, from 28.73% for manganese to 33.48% for lead. The influence of soil contamination with petrol on the chemical composition of the aerial parts of maize was weaker. Petrol had a stronger effect than the neutralizing materials only on two elements: iron (34.53%) and cobalt (42.91%), and dry matter yield aerial biomass of maize (80.41%).

## 4. Discussion

### 4.1. The Impact of Petrol Contamination on Plants

Petroleum substances affect the chemical composition of crops grown on polluted soils, modifying the content of macronutrients [54,55] and heavy metals in various plant organs [25,56,57], and in the soil itself [58].

Petroleum has a negative effect on the growth and development of plants [55,59]. This study demonstrated the stimulating influence of increasing petrol doses on the content of all heavy metals, except copper, in the aerial biomass of maize in the series without material amendments. Biomass maize yield was decreased. The lack of a significant effect was noted in the case of cadmium and zinc. An analogous effect of soil contamination with petrol (9 g kg^−1^) on the content of manganese, lead, and zinc in aerial parts of wheat was shown by Rusin et al. [60]. A positive correlation between soil contamination with petrol and the content of zinc, iron, and manganese in alfalfa plants leaves was also demonstrated by Martí et al. [61]. Gospodarek et al. [62], who tested the effect of a dose 6 g of petrol per 1 kg^−1^ d.m. of soil, observed reduced accumulation of copper, lead, zinc, nickel, iron, and manganese in aerial parts of *Vicia* *faba* beans. They also confirmed a statistically significant increase in the content of lead, cadmium, iron, and manganese in the roots of the tested plant relative to the control. Petroleum derivatives (e.g., heavy fuel spill) cause a rise in the soil content of cadmium, lead, copper, and manganese, leading to their higher mobility and bioavailability to plants [63,64]. Gospodarek et al. [55] observed an increase of some heavy metals (zinc, lead, and cadmium) in *Vicia faba* after contamination with petrol. Petroleum derived substances increased the zinc content in the leaves of some plant species (*Vernonia amygdelina*, *Talinum triangulare*, *Manihot esculenta*, and *Xanthotosoma sagittifolium*) [65]. This impact can partly explain the elevated content of lead and manganese in aerial parts of maize observed in our study. Rusin et al. [29] carried out an experiment, in which they tested the effect of soil pollution with a dose of petrol equal 6 g kg^−1^ and observed an increased accumulation of cadmium in wheat with the simultaneous decrease in the plant’s content of zinc. However, they did not note any significant changes in the content of copper. The results reported by these authors are different from the ones obtained in our experiment, which may have been caused by the differences in the tested soil contamination degree and the different test plants. Such dependences are confirmed by Gospodarek and Nadgórska-Socha [66].

### 4.2. The Impact of Materials Application on Plants on Soil Contaminated with Petrol

In response to the growing environmental threat, such as the contamination with petroleum substances, several technologies have been developed for remediation of anthropogenically polluted soils. An example is phytostabilisation, where soil amendments are used to depress the bioavailability and mobility of heavy metals and other pollutants [33,67,68]. The most popular soil amendments are phosphorus and calcium compounds, ashes, natural and synthetic aluminosilicates, and different forms of organic matter [68]. These materials have very good stabilising properties, in addition to which, they improve soil’s physicochemical characteristics and fertility [69,70].

Modified (organophilic) bentonites can be used in the process of reclamation of degraded soils, where they play a role of an adsorbent of aromatic organic compounds [71,72]. As demonstrated by Bertagnolli and da Silva [73], they are able to remove from 50% to 60% of BTEX compounds (benzene, toluene, ethylbenzene, and xylene), which are the most toxic components of petrol that contaminate soil. Shackelford and Jefferis [74] noted a positive effect of bentonite and calcium oxide on properties of soil polluted with petrol. Both neutralising materials tended to cause an increase in soil pH, total exchangeable bases, exchange capacity, and in base saturation, while leading to a decrease in hydrolytic acidity. The usefulness of bentonite and calcium oxide in the remediation of soil polluted with petrol, and their reducing influence on the content of polycyclic aromatic hydrocarbons has been shown by Wyszkowski and Ziółkowska [75]. The positive effect of calcium oxide on properties of contaminated soil, including the content of available forms of macro- and micronutrients and their uptake by plants, has been verified by other researchers [32,74].

Remediation of soil with compost and mineral materials tested in this study has contributed to the significant reduction in the content of manganese in the crop grown in all the experimental treatments. Similar results were obtained by Wyszkowski and Sivitskaya [76], who observed a decrease in the content of manganese in maize biomass by 68% and 44% following the application of respectively bentonite and calcium oxide. In the same experiment, the authors noted a significant rise in the content of lead and chromium, which partly agrees with the results of the current experiment.

The positive impact of compost and calcium oxide consisting of the limited uptake of zinc and lead by white lupine and on the yields of this crop has been confirmed by Castaldi et al. [77]. Wyszkowski and Ziółkowska [28] proved that the incorporation of bentonite, calcium oxide, and compost to soil improved the yields of crops, where bentonite had the best effect on spring oilseed rape while compost was most beneficial to oats. The same authors, in another experiment [7], demonstrated that compost and calcium oxide had a positive influence on the yield of yellow lupine (main crop) but did not cause any significant changes in yields of maize (catch crop).

In this experiment, the strongest effect on the chemical composition of maize was produced by calcium oxide. It resulted in a considerable reduction in the content of manganese as well as lead, zinc, and iron. Similar conclusions were drawn by Kosiorek and Wyszkowski [78], who found that the content of heavy metals in aerial parts of spring barley and white mustard depended most profoundly on the soil application of calcium oxide. The mentioned authors demonstrated a significant decrease in the content of manganese in aerial parts of barley (by 56%) and white mustard (by 83%) grown on cobalt-polluted soil after the soil application of calcium oxide. The same researchers noted the reducing effect of calcium oxide on the content of zinc, copper, and iron in the tested crops. The depressed translocation of manganese to aerial plant organs could be a consequence of the fact that the Mn^2+^ ion has similar properties to those of alkaline ions, like Ca^2+^, which induces antagonistic relations between the absorption of both ions [79].

The enrichment of soil with calcium oxide in an experiment conducted by Radziemska et al. [80] decreased the content of copper, zinc, and nickel in the aerial biomass of maize. Nagiel and Szulc [81] also confirmed the usefulness of soil liming in the immobilisation of heavy metals. They demonstrated that soil liming improved the yield of spring wheat as well as considerably reducing the uptake of cadmium and its content in the straw and grain of this crop. Tlustoš et al. [82] showed that the addition of lime to soil decreased the content of available forms of lead, zinc, and cadmium in the soil solution, hence limiting their translocation to aerial organs of plants. This is in agreement with the results obtained in our experiment in the series with the addition of calcium oxide.

The immobilising effect of alkaline compounds, such as calcium oxide, towards the bioavailable forms of heavy metals is associated with such changes as an increase in the amount of negatively charged soil particles, formation of hydroxyl compounds, with strong sorption properties, precipitation of elements in the form of hydroxides or carbonates, and their sequestration due to the higher microbial activity [83,84]. The application of calcium oxide has another positive effect in that it restores the biological balance of soil polluted with crude oil derivatives. As mentioned by Wyszkowska and Wyszkowski [85], compared to bentonite or compost, soil liming stimulated the activity of dehydrogenases, ureases, and alkaline phosphatase, and the process of nitrification while decreasing the activity of only acid phosphatase.

Measures taken to immobilise heavy metals in the soil sorption complex so as to prevent their translocation to further food chain links involving animals and humans seem to be the most appropriate approach to the reclamation of polluted soils. These are relatively inexpensive and technically easy methods. However, their outcome depends on the type of a pollution alleviating substance applied, properties of the soil and degree of soil contamination. It, therefore, seems proper to continue the research in this scope, in order to ensure environmental safety.

## 5. Conclusions

The contamination of soil with petrol and the application of compost and mineral materials to soil had a significant influence on the content of heavy metals in aerial parts and biomass yield of maize. The content of all heavy metals, except copper, in the aerial biomass of maize was positively correlated, but biomass yield negatively correlated, with the increasing doses of petrol. The highest increase in the content of heavy metals was noted for chromium and manganese.

All the three materials added to soil in order to neutralise the effect of petrol on the chemical composition of the tested plant had a significant and differentiating effect on the content of heavy metals and biomass yield of maize. They contributed to the increased accumulation of cadmium, chromium, copper, and cobalt in the aerial biomass of maize. The reducing effect of most of these materials was detected only in the case of manganese.

In comparison with the control series (without neutralising materials), the application of calcium oxide proved to be most effective. It had the most evident influence on the chemical composition of maize, limiting the accumulation of lead, zinc, manganese, and iron, and increasing biomass yield. However, the positive effect of calcium oxide on the maize biomass is greater than the negative effect.

The material amendments application can be a good and effective method in reclamation of soils contaminated with small doses of petrol.

## Figures and Tables

**Table 1:** Total content of heavy metals in soil and material amendments (mg kg^−1^ d.m.).

Element	Soil	Compost	Bentonite	Calcium Oxide
Cd	0.224	0.058	0.298	3.487
Pb	15.63	1.86	9.78	2.92
Cr	13.42	1.24	2.82	3.36
Ni	14.78	0.49	2.44	3.54
Zn	29.16	32.86	14.44	4.36
Cu	2.69	39.56	12.92	2.28
Mn	253.6	54.4	147.5	158.3
Fe	8256	229	4236	424
Co	3.44	0.49	0.30	1.73

**Table 2:** Dry matter yield of aerial parts of maize—*Zea mays* L. (averages ± standard deviation, g kg^−1^ d.m.).

Material	Petrol Dose (cm^3^ kg^−1^ d.m. of Soil)				Average
	0	2.5	5	10	
Without amendments	14.54 (±0.79) *^ab^*	12.07 (±0.63) *^cde^*	10.79 (±0.46) *^d–f^*	8.16 (±0.26) *^h^*	11.39 *^A^*
Compost	14.61 (±0.31) *^ab^*	12.24 (±0.74) *^cd^*	10.73 (±0.54) *^d–g^*	8.90 (±0.91) *^gh^*	11.62 *^AB^*
Bentonite	14.60 (±0.47) *^ab^*	11.47 (±0.77) *^c–f^*	11.20 (±0.50) *^c–f^*	10.21 (±0.75) *^e–g^*	12.12 *^AB^*
CaO	15.85 (±0.67) *^a^*	13.00 (±0.44) *^bc^*	11.98 (±0.76) *^c–e^*	10.05 (±0.36) *^fg^*	12.72 *^B^*

Values denoted by the different letters are significantly different at *p* ≤ 0.01: *^A,B^* for petrol dose and *^a^*^–*h*^ for interaction between petrol dose and material amendments (Anova, Tukey’s HSD test).

**Table 3:** Content of cadmium, lead, chromium, nickel, zinc, and copper in aerial parts of maize—*Zea mays* L. (averages ± standard deviation, mg kg^−1^ d.m.).

Material	Petrol Dose (cm^3^ kg^−1^ d.m. of Soil)				Average
	0	2.5	5	10	
Cadmium (Cd)					
Without amendments	0.093 (±0.001) *^a^*	0.094 (±0.001) *^ab^*	0.095 (±0.001) *^ab^*	0.096 (±0.003) *^a–c^*	0.095*^A^*
Compost	0.095 (±0.001) *^ab^*	0.096 (±0.000) *^a–c^*	0.097 (±0.001) *^a–c^*	0.097 (±0.001) *^a–c^*	0.096*^A^*
Bentonite	0.098 (±0.001) *^a–c^*	0.098 (±0.002) *^a–c^*	0.101 (±0.004) *^a–c^*	0.101 (±0.002) *^bc^*	0.100*^B^*
CaO	0.099 (±0.002) *^a–c^*	0.102 (±0.001) *^bc^*	0.100 (±0.001) *^a–c^*	0.103 (±0.001) *^c^*	0.101*^B^*
Lead (Pb)					
Without amendments	0.450 (±0.014) *^a–c^*	0.460 (±0.016) *^a–c^*	0.510 (±0.014) *^b–d^*	0.620 (±0.017) *^d^*	0.510*^A^*
Compost	0.450 (±0.014) *^a–c^*	0.580 (±0.008) *^cd^*	0.600 (±0.028) *^d^*	0.525 (±0.017) *^b–d^*	0.539*^A^*
Bentonite	0.520 (±0.008) *^b–d^*	0.531 (±0.022) *^b–d^*	0.500 (±0.009) *^a–d^*	0.443 (±0.034) *^ab^*	0.498*^A^*
CaO	0.377 (±0.002) *^a^*	0.378 (±0.023) *^a^*	0.452 (±0.016) *^a–c^*	0.503 (±0.022) *^a–d^*	0.427*^B^*
Chromium (Cr)					
Without amendments	1.200 (±0.094) *^a^*	2.833 (±0.047) *^ab^*	3.433 (±0.041) *^b^*	3.800 (±0.034) *^bc^*	2.816*^A^*
Compost	3.000 (±0.074) *^ab^*	4.433 (±0.037) *^b–d^*	4.233 (±0.052) *^b–d^*	5.399 (±0.084) *^c–e^*	4.266*^B^*
Bentonite	5.299 (±0.047) *^c–e^*	5.966 (±0.041) *^d–f^*	7.033 (±0.036) *^e–g^*	7.599 (±0.084) *^f–h^*	6.474*^C^*
CaO	6.466 (±0.028) *^ef^*	7.333 (±0.043) *^f–h^*	8.399 (±0.057) *^gh^*	9.066 (±0.028) *^h^*	7.816*^D^*
Nickel (Ni)					
Without amendments	1.178 (±0.114) *^ab^*	1.517 (±0.124) *^a–d^*	1.680 (±0.097) *^a–e^*	1.913 (±0.195) *^b–f^*	1.572*^A^*
Compost	1.027 (±0.067) *^a^*	1.365 (±0.082) *^a–c^*	1.726 (±0.079) *^a–f^*	1.82 (±0.099) *^b–f^*	1.484*^A^*
Bentonite	1.703 (±0.089) *^a–f^*	2.450 (±0.099) *^f^*	2.275 (±0.026) *^ef^*	1.785 (±0.115) *^b–f^*	2.053*^B^*
CaO	1.540 (±0.033) *^a–e^*	2.158 (±0.082) *^d–f^*	2.030 (±0.084) *^c–f^*	1.890 (±0.114) *^b–f^*	1.904*^B^*
Zinc (Zn)					
Without amendments	13.33 (±0.03) *^ab^*	13.43 (±0.11) *^ab^*	14.62 (±0.24) *^ab^*	14.69 (±0.15) *^ab^*	14.02*^A^*
Compost	13.23 (±0.43) *^ab^*	15.08 (±0.35) *^ab^*	16.04 (±0.28) *^ab^*	14.10 (±0.15) *^ab^*	14.61*^AB^*
Bentonite	16.78 (±0.63) *^ab^*	18.90 (±0.37) *^b^*	16.88 (±0.27) *^ab^*	14.96 (±0.16) *^ab^*	16.88*^B^*
CaO	11.35 (±0.29) *^a^*	14.16 (±0.09) *^ab^*	14.39 (±0.03) *^ab^*	11.37 (±0.26) ^a^	12.82*^A^*
Copper (Cu)					
Without amendments	1.000 (±0.084) *^a^*	1.333 (±0.093) *^ab^*	0.750 (±0.058) *^a^*	0.667 (±0.036) *^a^*	0.937*^A^*
Compost	1.667 (±0.011) *^a–c^*	2.083 (±0.098) *^a–c^*	3.133 (±0.024) *^b–d^*	3.750 (±0.082) *^cd^*	2.658*^B^*
Bentonite	4.833 (±0.084) *^d^*	7.916 (±0.123) *^e^*	10.499 (±0.287) *^f^*	10.832 (±0.236) *^fg^*	8.520*^C^*
CaO	6.999 (±0.087) *^e^*	12.332 (±0.122) *^fg^*	12.832 (±0.171) *^g^*	15.248 (±0.225) *^h^*	11.853*^D^*

Values denoted by the different letters are significantly different at *p* ≤ 0.01: *^A–D^* for petrol dose and *^a^*^–*h*^ for interaction between petrol dose and material amendments (Anova, Tukey’s HSD test).

**Table 4:** Content of manganese, iron and cobalt in aerial parts of maize—*Zea mays* L. (averages ± standard deviation, mg kg^−1^ d.m.).

Material	Petrol Dose (cm^3^ kg^−1^ d.m. of Soil)				Average
	0	2.5	5	10	
Manganese (Mn)					
Without amendments	29.05 (±1.15) *^bc^*	30.45 (±1.24) *^b–d^*	38.96 (±0.99) *^de^*	85.74 (±2.27) *^f^*	46.05*^A^*
Compost	27.41 (±0.49) *^a–c^*	28.58 (±0.16) *^a–c^*	30.45 (±0.82) *^b–d^*	41.76 (±0.24) *^e^*	32.05*^B^*
Bentonite	29.63 (±0.24) *^bc^*	34.53 (±0.24) *^c–e^*	30.56 (±0.24) *^b–d^*	30.20 (±1.16) *^b–d^*	31.23*^B^*
CaO	19.83 (±1.31) *^a^*	25.20 (±0.75) *^ab^*	27.18 (±0.49) *^abc^*	27.41 (±0.82) *^a–c^*	24.91*^C^*
Iron (Fe)					
Without amendments	31.40 (±0.62) *^ab^*	39.26 (±1.17) *^a–e^*	40.09 (±1.78) *^a–e^*	44.16 (±1.87) *^c–f^*	38.73*^A^*
Compost	29.61 (±1.31) *^a^*	35.19 (±1.33) *^a–c^*	37.01 (±1.79) *^a–c^*	34.87 (±1.25) *^a–c^*	34.17*^C^*
Bentonite	42.89 (±1.72) *^b–f^*	52.13 (±1.23) *^f^*	48.71 (±1.42) *^d–f^*	38.43 (±0.56) *^a–d^*	45.54*^B^*
CaO	33.16 (±1.65) *^a–c^*	50.23 (±1.27) *^ef^*	54.18 (±1.80) *^f^*	33.44 (±1.13) *^a–c^*	42.75*^AB^*
Cobalt (Co)					
Without amendments	0.320 (±0.014) *^a^*	0.352 (±0.011) *^ab^*	0.412 (±0.004) *^a–c^*	0.457 (±0.011) *^a–c^*	0.386*^A^*
Compost	0.415 (±0.014) *^a–c^*	0.432 (±0.018) *^a–c^*	0.437 (±0.025) *^a–c^*	0.560 (±0.007) *^c^*	0.461*^B^*
Bentonite	0.417 (±0.018) *^a–c^*	0.470 (±0.014) *^a–c^*	0.497 (±0.004) *^bc^*	0.580 (±0.017) *^c^*	0.491*^B^*
CaO	0.475 (±0.009) *^a–c^*	0.487 (±0.013) *^a–c^*	0.507 (±0.012) *^bc^*	0.540 (±0.017) *^c^*	0.502*^B^*

Values denoted by the different letters are significantly different at *p* ≤ 0.01: ^A–*C*^ for petrol dose and ^a–*f*^ for interaction between petrol dose and material amendments (Anova, Tukey’s HSD test).

## Data Availability

Data are available by contacting the authors.
